# HistoML, a markup language for representation and exchange of histopathological features in pathology images

**DOI:** 10.1038/s41597-022-01505-0

**Published:** 2022-07-08

**Authors:** Peiliang Lou, Chunbao Wang, Ruifeng Guo, Lixia Yao, Guanjun Zhang, Jun Yang, Yong Yuan, Yuxin Dong, Zeyu Gao, Tieliang Gong, Chen Li

**Affiliations:** 1 School of Computer Science and Technology, Xi’an Jiaotong University, Xi’an, Shaanxi 710049 China; 2 Department of Pathology, The First Affiliated Hospital of Xi’an Jiaotong University, 277 West Yanta Road, Xi’an, Shaanxi China; 3 Division of Anatomic Pathology, Department of Laboratory Medicine and Pathology, Mayo Clinic, Rochester, Minnesota USA; 4 Department of Health Services Administration and Policy, Temple University, Philadelphia, PA USA; 5 Department of Pathology, The Second Affiliated Hospital of Xi’an Jiaotong University, No. 3, Shang Qin Road, Xi’an, Shaanxi China; 6 Department of Pathology, Shaanxi Provincial Tumor Hospital, Xi’an Jiaotong University, 309 Yanta West Road, Xi’an, Shaanxi China; 7 Key Laboratory of Intelligent Networks and Network Security (Xi’an Jiaotong University), Ministry of Education, Xi’an, Shaanxi 710049 China; 8 National Engineering Lab for Big Data Analytics, Xi’an Jiaotong University, Xi’an, Shaanxi 710049 China

**Keywords:** Research data, Medical research

## Abstract

The study of histopathological phenotypes is vital for cancer research and medicine as it links molecular mechanisms to disease prognosis. It typically involves integration of heterogenous histopathological features in whole-slide images (WSI) to objectively characterize a histopathological phenotype. However, the large-scale implementation of phenotype characterization has been hindered by the fragmentation of histopathological features, resulting from the lack of a standardized format and a controlled vocabulary for structured and unambiguous representation of semantics in WSIs. To fill this gap, we propose the Histopathology Markup Language (HistoML), a representation language along with a controlled vocabulary (Histopathology Ontology) based on Semantic Web technologies. Multiscale features within a WSI, from single-cell features to mesoscopic features, could be represented using HistoML which is a crucial step towards the goal of making WSIs findable, accessible, interoperable and reusable (FAIR). We pilot HistoML in representing WSIs of kidney cancer as well as thyroid carcinoma and exemplify the uses of HistoML representations in semantic queries to demonstrate the potential of HistoML-powered applications for phenotype characterization.

## Introduction

The analysis of histopathological phenotypes plays a key role in cancer research and medicine; yet it remains a challenge to accurately define histopathological phenotypes due to highly heterogeneous and complex nature of tumor cells’ spatial distribution^1^. The recent progress of digital pathology and deep learning methods for image analysis facilitates large-scale extraction of histopathological features (e.g. cells, tissues, phenotypes) from whole-slide images (WSI) and improves the quantitative analysis techniques^2^. The integration of the various features as well as the analysis results sheds the light on objectively characterizing histopathological phenotypes. However, it lacks a standardized digital format and a well-defined controlled vocabulary for representing the semantics in WSIs following the FAIR (Findable, Accessible, Interoperable and Reusable) standards^3^. Consequently, fragmentation of the information hinders large-scale integrated analysis of histopathological phenotypes.

Histopathological phenotypes refer to the phenotypes of tissues observed microscopically by a pathologist from a biopsy or surgical specimen. The complexity and heterogeneity of histopathological phenotypes lie in not only diverse types of the individual components (e.g. cells, tissues, substances), but also in their morphologies, spatial arrangements (e.g. architectural patterns) and behaviors (e.g. invasion, extension). In view of this, it has been recognized that integrated analysis of histopathological features is the key to characterizing histopathological phenotypes^4^. As multi-scale histopathological features could be automatically extracted from WSIs using deep learning methods, some works have successfully applied this methodology to characterizing tumor-immune phenotypes^5,6^. However, the large-scale implementation of this methodology, for illuminating the intra-tumoral spatial heterogeneity, has so far remained elusive resulting from the lack of FAIR WSI datasets.

**Fig. 1:**
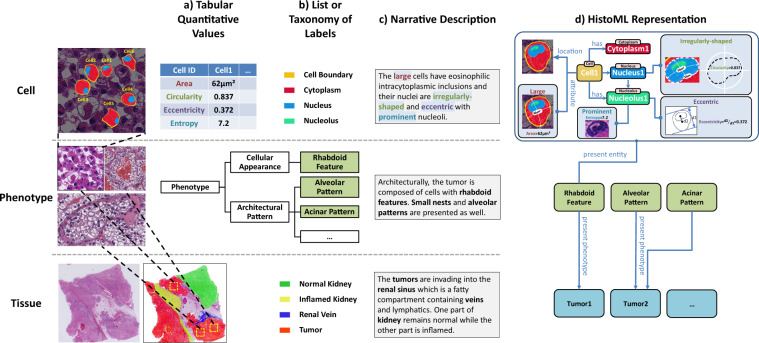
Exemplar representations of histopathological features using the previous representations approaches vs. HistoML representation. Shown histopathological features include tissue-level, phenotype-level, cellular-level features as well as quantitative analysis results. **(a–c)** Multi-scale histopathological features are currently described using different representation approaches by different groups. Quantitative analysis results are usually represented in tables; histopathological phenotypes, tissues and cells appeared in a whole-slide image (WSI) are represented using a list or taxonomy of labels while lacking a unified controlled vocabulary; more detailed descriptions of the intra-tumoral morphology rely on natural language which usually suffer from ambiguity, making natural language processing an error-prone process. **(d)** HistoML represents multi-scale histopathological features in a unified and machine-readable structure with their relationships to one another specified explicitly, providing a systems-level view of histopathological phenotypes.

The total volume of WSI data has entered a rapid growth phase, as exemplified by lymphoma^7^ and breast cancer^8^. In addition, numerous deep learning models for histopathological image analysis including segmentation, classification, detection and quantitative analysis of multi-scale histopathological features (e.g. tumor-level^9^, tissue-level^10^, phenotype-level^11^ and single cell-level^12^) provide large amounts of information about histopathological phenotypes. Unfortunately, the information is stored in different file formats (e.g. CSV, JSON, XML) and represented using custom representation approaches (Fig. 1a,b,c), which are broadly adopted by the information systems of hospitals, annotations of histopathology datasets, software tools and computational models (Table 1). These representations do not share a controlled vocabulary and suffer from inflexibility and ambiguity (Table 2), thereby resulting in a heterogeneous set of resources that are extremely difficult to combine and reuse. The desire to achieve integrated analysis of histopathological phenotypes calls for a semantic standard to produce large-scale FAIR WSI data^4^; yet it remains a challenge to comprehensively and accurately represent the rich meaning within WSIs in a standardized and machine-readable format.

**Table 1:** Different types of histopathological features within whole-slide images (WSI) and the representation approaches used to describe them.

Representation Approaches	Histopathological Features
List of labels	1. Different types of tissues and cells appeared in a WSI^10^
	2. Histopathological phenotypes in a WSI^11,44,45^
Taxonomy of labels	1. Different types of cells together with their locating tissues appeared in a WSI^12^
	2. Histopathological phenotypes in a WSI^46–48^
Narrative description	1. Morphologic characteristics of histopathological phenotypes^48,49^
Tabular Quantitative Values	1. Cell segmentations masks, spatial features like cell size and shape and subcellular morphological features^33,34^

**Table 2:** Inflexibility, ambiguity and inconsistency of the current representation approaches.

Representation Approaches	Problem
List of labels & Taxonomy of labels	1. List of labels is suitable to represent discrete histopathological features (e.g. different types of cells) in data while unable to represent their relationships (e.g. containment).
	2. Single-parent hierarchies and connections of taxonomy preclude description of the complex interrelationships between histopathological features.
	3. The information content could not be updated continuously and therefore unable to incorporate or exploit rapidly evolving histopathology knowledge.
	4. Use of subjective words and synonyms would make it hard for machines to compare and understand different features.
	5. Different groups use ad hoc controlled vocabularies having much inconsistency and cover only limited histopathological concepts.
Narrative description	Though easy to be obtained from pathology reports, it introduces great ambiguity into the representations, thereby preventing machines from comparing and understanding histopathological features.
Tabular Quantitative Values	Quantitative analysis results are suitable to objectively represent histopathological features and make them comparable while unable to represent their relationships (e.g. containment).

In this paper, we apply Semantic Web technologies (SW) to addressing this challenge by proposing the Histopathology Mark-up Language (HistoML), a representation language with a flexible syntax and extensible structure, along with a controlled vocabulary (Histopathology Ontology) to represent semantics in WSIs. The integration solutions enabled by SW have benefited many scientific fields, including system biology, integrative neuroscience, bio-pharmaceutics and translational medicine^13^. While some standards for highly multiplexed tissue images are underdeveloped^14^, HistoML is developed focusing on histopathology images; moreover, the technical features of SW make HistoML more advanced than the previous works. First, in addition to single-cell features, HistoML could further represent mesoscopic-scale characteristics of histopathological phenotypes within WSIs such as spatial arrangement of tissues which information is challenging to represent and consequently lacking in many publicly accessible atlases of human tissues and tumors^15^. Second, as a mark-up language based on Web Ontology Language (OWL), HistoML could integrate various histopathological features and analysis results of WSIs, fragmented in the previous representations, into a coherent representation (Fig. 1d), with their relationships to one another specified explicitly, providing a systems-level view of histopathological phenotypes. Third, we propose Histopathology Ontology which has a broad coverage of histopathological concepts by reusing a number of widely-applied ontological resources relevant to histopathology. To validate our work, we pilot HistoML in representing semantics in WSIs of kidney cancer and thyroid carcinoma. Furthermore, we exemplify the uses of HistoML representations in semantic queries to demonstrate the potential of HistoML-powered applications for phenotype characterization.

## Results

### HistoML Project

Developing a standardized format for representing semantics in histopathology data is challenging owing to the heterogeneity of histopathological features and their diverse uses in cancer research and medicine. For HistoML to be successful, it must satisfy a majority of technical and practical needs of pathologists, biomedical researchers, IT experts and artificial intelligence (AI) scientists in order to be embraced by the community. We organize the HistoML project with engaging the community and build a consensus among different participants in mind. Additionally, we strive to follow the FAIR standards and seek to avoid many problems (Table 2) of the existing representation approaches. Therefore, we set up the following principles to steer HistoML toward those aims.

The language should

- be free and open to allow free use by the community;
- support representation of diverse histopathological features;
- be syntactically and semantically consistent and unambiguous;
- ensure the semantic interoperability of the representations constructed by different groups;
- support continuous integration of new histopathological features as histopathology knowledge evolves while the existing representations should remain compatible and usable;
- support the automated accessing and querying of the represented features in addition to storage and dissemination by software and computational models;

As designing a perfect and complete language from the beginning is impossible, HistoML development is envisioned to proceed in stages. Major editions of HistoML are termed ‘levels’ with each higher HistoML level supporting representation of more histopathological features compared to the levels below it by adding additional structures and facilities. Moreover, through mapping all of the constructs from the previous level to the next level, different levels of HistoML and the corresponding representations would remain compatible and usable.

### Overview of HistoML Level 1

In this paper, we propose HistoML Level 1, which is the first implementation of these principles, with the aim to represent histopathological features in tumor including histopathological phenotypes, their individual components as well as their properties, relationships and behaviors.

**Fig. 2:**
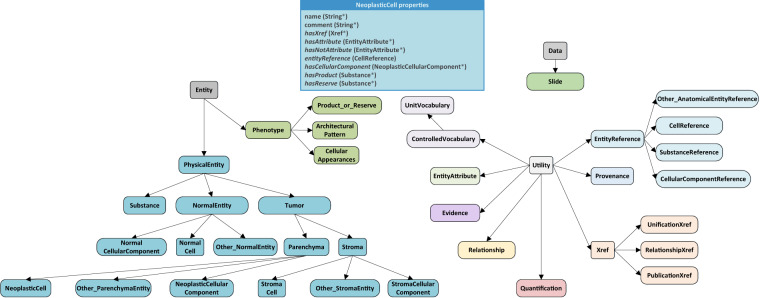
High-level view of the HistoML Level 1. HistoML classes are shown as boxes and the arrows represent subclass relationships. An individual of **NeoplasticCell** class is shown as an example at the top. The object properties are italicized while the datatype properties are not and the asterisks indicate that multiple values for the property are allowed. Refer to HistoML Level 1 ontology specification and documentation at https://histoml.com/ for full details of all the classes and properties.

The structure (i.e. schema) of HistoML is implemented as an ontology^16^ (Fig. 2) which has been widely used to structurally represent knowledge regarding life sciences^17^. **Entity**, **Utility** and **Data** are the three root classes of HistoML in which **Entity** includes phenotypes, physical entities, while **Utility** includes classes for annotating **Entity** and its subclasses; **Data** includes classes for storing metadata of the raw histopathology data (e.g. height, width, magnification of a WSI). The semantics of a WSI are represented as individuals of HistoML classes (an individual of **NeoplasticCell** class is shown in Fig. 2), in which the object properties describe the relationships between HistoML individuals and datatype properties store metadata such as names or numerical values. A HistoML representation of a WSI mainly consists of individuals of lists of these classes:

**PhysicalEntity**: A description of a microscopically observable entity in the WSI (e.g. cell, tissue, chemical substance).

**EntityAttribute**: An attribute of a physical entity that can be changed while the entity still retains its biological identity such as size, shape, length.

**Quantification**: A quantity used to quantitatively describe the attribute of an entity by providing the absolute amount or the numerical range of the attribute.

**Phenotype**: A description of microscopic physical changes in the WSI that prompt pathologists to look more closely, typically the ones suggesting malignancy. A phenotype composes of one or more physical entities which might have relationships with each other (e.g. containment) or behaviours (e.g. growth, invasion).

The syntax of HistoML is based on OWL in order to simplify the use of HistoML representations by taking advantage of existing software tools for editing, transmitting, querying, reasoning about and visualizing OWL. A software or a computational model can also read in a WSI expressed in HistoML and translate it into its own internal format for further analysis.

### Components of HistoML

**Fig. 3:**
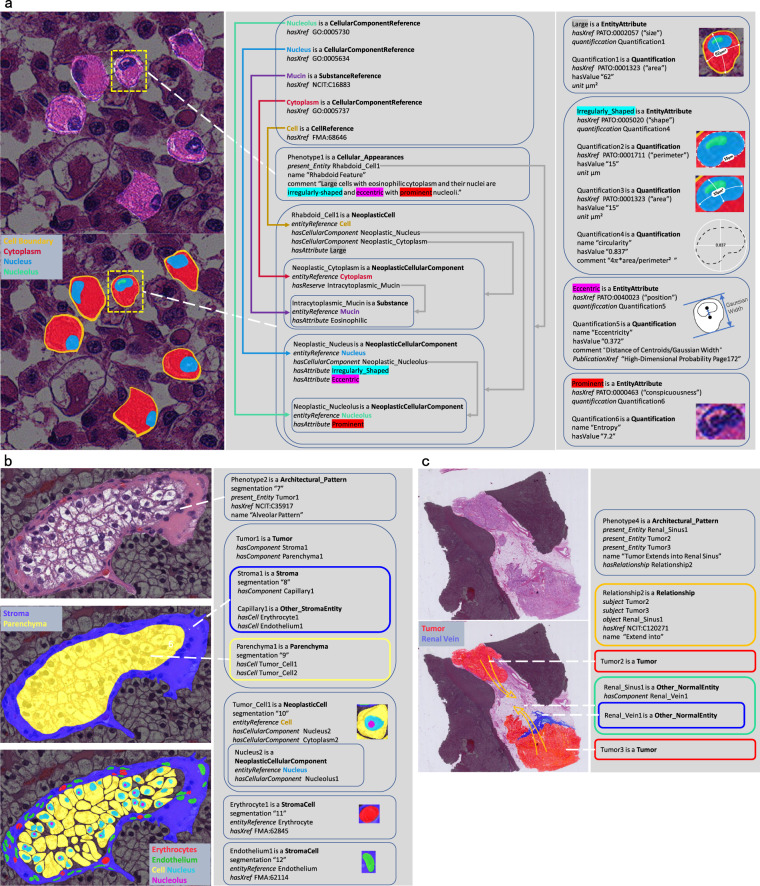
Three exemplar HistoML representations of histopathological phenotypes. An individual of HistoML class is shown as a rounded rectangle. HistoML class names are highlighted in bold, HistoML object properties are italicized while the datatype properties are not. **(a)** An example of **Cellular_Appearances** representing rhabdoid cells in RCC (renal cell carcinoma). On the left are the original image and the one that has been manually annotated by an expert pathologist. In the middle are the representations of the phenotype as well as its individual components. On the right are the representations of their properties using **EntityAttribute** and **Quantification**. **(b)** An example of **Architectural_Pattern** representing an alveolar pattern of ccRCC (clear cell renal cell carcinoma). On the left are the original image and two that have been manually annotated and on the right is the representation. **(c)** An example of representing tumors extending into renal sinus. The green rectangle corresponds to the left space of the renal sinus other than the space occupied by the tumors, the yellow arrows indicate the directions of the tumor extension. The complete HistoML representations of these three examples are available at https://histoml.com/ which contain descriptions of all the individual components, while space limitations permit us to show only a few in this figure.

In the following sections, we describe the various classes of HistoML with the help of three exemplar HistoML representations which describe three typical histopathological phenotypes of kidney cancer, including the rhabdoid feature, the alveolar pattern and the tumors’ extension into renal sinus. The raw images as well as the annotated ones of these phenotypes are shown in Fig. 3 and their textual definitions are provided in Table 3. A more realistic example, in which the histopathological features in a WSI of papillary thyroid carcinoma are represented using HistoML, is shown in Supplementary Figs. 1 and 2. These examples illustrate one application of HistoML, but users could customize HistoML representations to describe histopathological phenotypes of other neoplastic diseases. Space constraints prevent us from giving a detailed description of HistoML here; the full definition is available in the specification available from https://histoml.com/.

**Table 3:** Definitions of the three represented histopathological phenotypes.

Histopathological Phenotype	Definition
Rhabdoid Feature	The rhabdoid cells are large neoplastic cells with eosinophilic intracytoplasmic inclusions and their nuclei are irregularly-shaped and eccentric with prominent nucleoli^36^.
Alveolar Pattern	An alveolar pattern is morphologically similar to alveoli or little cells, sacs or nests in which no rounded luminal space is present^31,36^.
Tumors’ Extension into Renal Sinus	The spread or migration of cancer cells into renal sinus which is a fatty compartment containing veins and lymphatics^50^.

#### PhysicalEntity

The **PhysicalEntity** class in HistoML is used to represent microscopically observable entities in a slide. According to their different types, **PhysicalEntity** has several subclasses covering cells, cellular components, substances (e.g. product or reserve of a cell), tissues and other anatomical structures (e.g. a cavity or duct). It’s further categorized into **NormalEntity** and **Tumor** based on whether an entity is neoplastic or induced by tumor or not (e.g. blood vessels induced by tumor belong to **Tumor** instead of **NormalEntity**). An individual of **PhysicalEntity** is shown in Fig. 3a referred to as “Rhabdoid_Cell1”.

**PhysicalEntity** has four main object properties which are *entityReference*, *hasAttribute*, *hasProduct* and *hasComponent*. Firstly, there are often many entities all of the same kind in a WSI; in order to create different forms of a generic entity without duplicating information common to all the forms, the generic entity is defined using **EntityReference**, while its diverse forms are defined using **PhysicalEntity**; *entityReference* is used to link them together. Secondly, *hasAttribute* is used to define the attributes of a physical entity; for example, shape, size or other chemical attributes such as eosinophilic. A set of attributes helps define the state of an entity. Thirdly, a good number of materials within cells, known as the granular cytoplasmic inclusions such as mucin, acid, glycogen and starch, are also microscopically-observable and they are the product or reserve of these cells; *hasProduct* and *hasReserve* are used to represent this information. Last but not least, *hasComponent* is used to express containment relationships between **PhysicalEntity** which has three child properties including *hasCell*, *hasCellularComponent* and *hasAnatomicalEntity*.

#### EntityAttribute and Quantification

Pathologists usually assess morphological characteristics of physical entities subjectively and use unspecific words (e.g. “atypical”, “large”) to describe them. It brings much ambiguity for objectively characterizing histopathological phenotypes. For example, “atypical nuclear change” could be confusing since it might mean irregularity in the nuclear size or shape; moreover a “large” kidney tumor cell is usually smaller than a “small” liver tumor cell. To avoid this issue, HistoML uses **EntityAttribute** to specify what attribute is “atypical” by providing their semantic cross-references to the controlled vocabulary such as The Phenotype And Trait Ontology (PATO) through an object property named *hasXref*. Additionally, as the advances of quantitative histomorphometry (QH) facilitate quantitatively assessing tissue morphology and architecture^18^, HistoML uses **Quantification** to illustrate how “atypical” the attribute is by incorporating QH measurements. For example, as Fig. 3a shows, “large” could be defined by area, “irregularly-shaped” could be defined by circularity etc. **Quantification** could provide values of the measurements, the unit definitions as well as the formulas regarding how to calculate the measurements. Furthermore, if available, the link of the source material proposing the measurement (e.g. Gaussian Width^19^) could be added to **Quantification** for further validation. In combination of **EntityAttribute** and **Quantification**, it is easy to objectively compare morphological characteristics of the physical entities.

#### Phenotype

**Phenotype** class has three subclasses which are **Cellular_Appearances**, **Product_or_Reserve** and **Architectural_Pattern**, covering different levels of phenotypes that could appear in a WSI. HistoML represents a phenotype in which each of the individual components is described, including their properties, relationships and behaviors.

**Cellular_Appearances** and **Product_or_Reserve** are used to describe a physical change of a single cell or a subcellular structure usually observed microscopically at high magnification. The comprehensive and quantitative description of the physical entity’s morphology is the key to objectively characterize these two types of phenotypes. An example of **Cellular_Appearances** representing the rhabdoid feature in RCC (renal cell carcinoma) is shown in Fig. 3a. According to the definition of this phenotype (Table 3), there are four main characteristics differentiating rhabdoid cells from others which are the size of the cell, the shape of the nucleus, the position of the nucleus within the cell as well as the prominence of the nucleolus. We represent this phenotype by defining these characteristics using **EntityAttribute** and their owner physical entities using **PhysicalEntity**; moreover, we use four parameters to quantify the characteristics including area, circularity, eccentricity and image entropy. *present_Entity* is used to link the phenotype to its component.

**Architectural_Pattern** is used to describe histologic patterns of cell populations and tumor behaviors which are usually observed microscopically at medium and low magnification. The spatial arrangement of the components is the key to characterize a histologic pattern. HistoML represents the pattern by specifying the components, their containment relationships and providing their segmentation masks to make the components spatially-resolved. An example of **Architectural_Pattern** representing the alveolar pattern of ccRCC (clear cell renal cell carcinoma) is shown in Fig. 3b. According to its definition, it is a neoplastic area which consists of a stroma and parenchyma; the stroma is basically a capillary containing erythrocytes and endothelia and the parenchyma is full of neoplastic cells. We represent this phenotype by specifying all of these physical entities using **PhysicalEntity** as well as their containment relationships through *hasComponent*. As for the spatial arrangement information that the parenchyma is surrounded by the capillary and the distribution of the neoplastic cells in the parenchyma, we represent it by providing the locations of these physical entities in the WSI (e.g. positions of the pixels) through a datatype property named ‘segmentation’, which stores the identifiers of the segmentations or the annotation masks. ‘segmentation’ links the semantic representations of histopathological features with their positions in a WSI, thereby making it possible for scientists to assess the features themselves and keep track of the tissue context and their spatial attributes. Another example of **Architectural_Pattern** representing a tumor behavior, which is a tumor extension into renal sinus, is shown in Fig. 3c. Tumor is in motion within the human body while a WSI is only one frame of the movement. HistoML describes the movement by specifying the types of the movement, the moving object and the subject the movement towards using **Relationship**.

In summary, compared with the current representation approaches, HistoML could provide a far more comprehensive description of a histopathological phenotype by interrelating layers of information in a WSI, which paves the way for integrated analysis of intra-tumoral spatial heterogeneity.

### Histopathology Ontology

To ensure semantic interoperability between HistoML representations constructed by different groups, it is vital to create a controlled and shared vocabulary for achieving consistency in preferred terms and the assignment of the same terms to similar content. Therefore, we constructed Histopathology Ontology, with the goal of covering all information HistoML could represent. Histopathology Ontology is applicable to human pathology, especially for tumor pathology. In HistoML, **Xref** class and *hasXref* are used to map HistoML representations to the controlled vocabulary.

We build Histopathology Ontology by reusing a number of widely-applied ontological resources relevant to histopathology. For example, histopathological phenotypes are described using National Cancer institute’s Thesaurus (NCIt)^20^ and Cellular Microscopy Phenotype Ontology (CMPO)^21^; their individual components are described using Foundational Model of Anatomy Ontology (FMA)^22^ and Gene Ontology (GO)^23^; their properties are described using The Phenotype And Trait Ontology (PATO)^24^; units of quantification are described using the Units Ontology (UO)^25^ etc. Most of these reference ontologies are part of the Open Biological and Biomedical Ontologies (OBO) Foundry^26^ which are designed to be reused by multiple groups and stakeholders. Mapping to these terms, HistoML representations can be easily integrated not only with each other, but also into the ecosystem of other datasets that are annotated using these ontologies. Furthermore, implemented as an ontology, the structure of Histopathology Ontology is multi-parentage (i.e. one term could have multiple parents); as a result, compared with the ad hoc controlled vocabularies organized in list or in a single-parent hierarchy, it’s easier to continuously generate and merge new terms to Histopathology Ontology to exploit evolving histopathology knowledge. Histopathology Ontology is freely available at https://histoml.com/.

### Use of Histopathological Features Encoded in HistoML

In addition to the broad coverage of histopathological features, HistoML representations are highly structured that users could easily access various features through SPARQL queries; moreover, the query results could be used to enable advanced analysis of the features. In this section, we demonstrate that histopathological features represented in HistoML can be queried using SPARQL. Furthermore, we show how the queried results could be used for phenotype characterization.

**Fig. 4:**
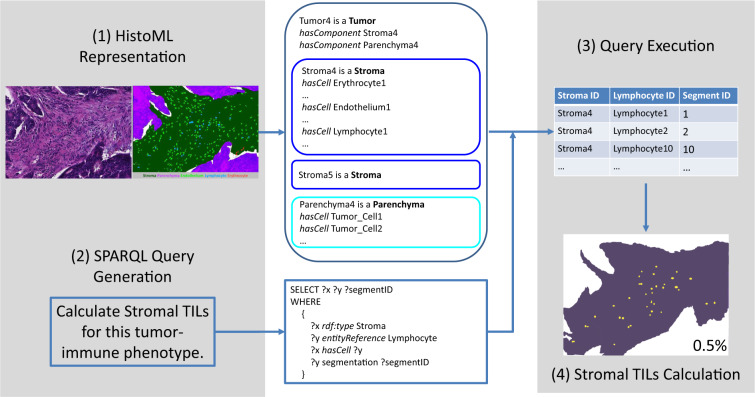
Quantitatively characterizing a tumor-immune phenotype of breast cancer by calculating the stromal tumor-infiltrating lymphocytes (TILs). A raw image and an annotated image of a tumor-immune phenotype of breast cancer are shown in the top-left corner. In the annotated image which is on the right of the raw image, the parenchyma and the stroma area within which there are many lymphocytes, endothelia, erythrocytes, are annotated. Firstly, the image of this phenotype is represented using HistoML and a SPARQL query is generated. Secondly, we could obtain the stromal components, the infiltrated lymphocytes as well as their corresponding pixels in the image by running the SPARQL query on the HistoML representation. Thirdly, we could obtain Stromal TILs of this phenotype by calculating the area of each lymphocyte and stromal component.

Figure 4 demonstrates one SPARQL query over a HistoML representation. The characteristics of a tumor-immune phenotype of breast cancer are represented using HistoML and a few lines of SPARQL query code are able to obtain the stromal component, the infiltrated lymphocytes (i.e. the lymphocytes within the stromal component) as well as their corresponding segmentations in the raw image. The query results can be further used to calculate the stromal tumor-infiltrating lymphocytes (TILs), a crucial parameter for characterizing tumor-immune phenotypes^27^. As the areas of the stroma and the infiltrated lymphocytes could be obtained through their segmentations, the value of the stromal TILS is the fraction of the stroma covered by the lymphocytes. The source code of this use case is available at https://github.com/Peiliang/HistoML.

## Discussion

**Fig. 5:**
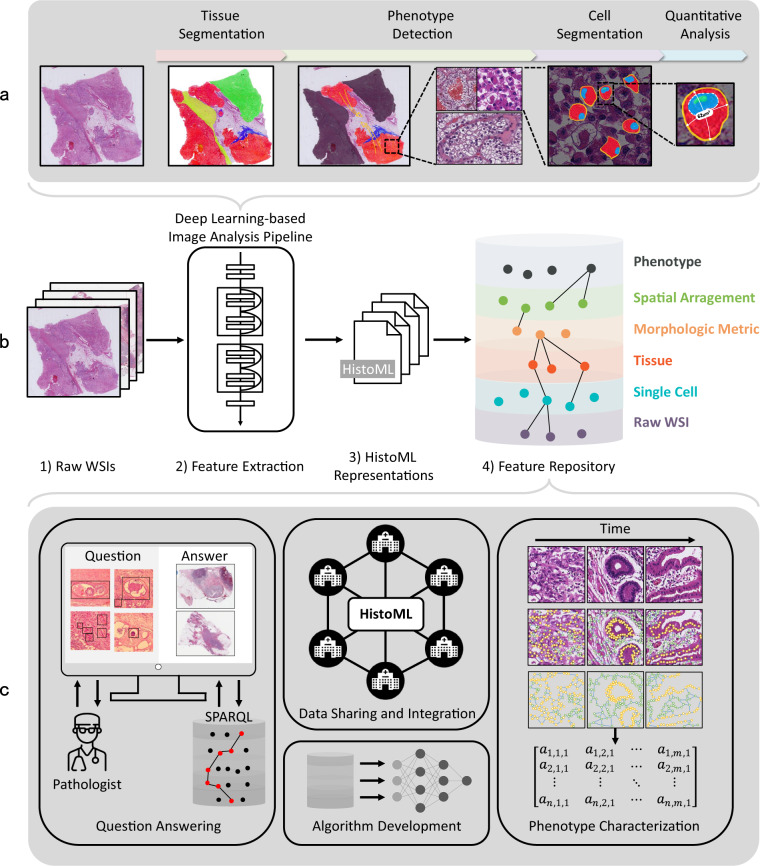
Future Applications of HistoML. **(a)** A deep-learning based histopathological image analysis pipeline which consists of segmentation, detection and quantitative analysis of multi-scale histopathological features including tissue-level, phenotype-level and single cell-level features. **(b)** A flow diagram of producing HistoML representations of WSI data and constructing a feature repository of histopathology. **(c)** Potential applications of the feature repository including sharing and integrating WSI data from multiple sources, developing a question-answering system, training image analysis algorithms and characterizing phenotypes using mathematical models.

HistoML is a machine-readable format with a flexible syntax and an extensible structure; moreover, HistoML representations cover various histopathological features in WSI data as well as the metadata. In combination of these characteristics, HistoML could facilitate several applications as Fig. 5 shows. Firstly, HistoML and Histopathology Ontology could be used as a shared language and controlled vocabulary of WSI data which would reduce the number of translations required to exchange information between multiple sources. Furthermore, it facilitates integration of WSI data by consistently and comprehensively representing heterogenous histopathological features extracted from WSIs by deep learning methods, as Fig. 5a shows. As a result, it paves the way to construct a feature repository of histopathology (i.e. knowledge base) in addition to the raw data repository (e.g. The Cancer Genome Atlas^28^) as Fig. 5b shows. This feature repository would be an important resource to pathology, as some of the best practices of FAIRness to the life sciences, such as UniProt^29^ and BioModels^30^.

HistoML representations of WSI data could contribute to integrated analysis of histopathological phenotypes, as Fig. 5c shows. Firstly, HistoML makes it easy for researchers and pathologists to implement multi-dimensional queries of histopathological features using SPARQL. Interfacing with such service, the feature repository could answer key questions about the intra-tumoral spatial heterogeneity. For example, when a pathologist observes a novel combination of histopathological features in his cases that he has never been observed before which might indicate a new disease subtype, he could test whether this observation exists in the cases of other medical centers by creating just a simple SPARQL query. However, this process of validation, as a crucial step for precise classification of patients, traditionally takes years as the evidential cases are obtained mainly from medical publications and thus accumulate slowly; for example, it took ten years to involve acquired cystic RCC in the WHO Classification of kidney tumors^31^ since its pathologically unique features had been firstly discovered^32^. In contrast, searching on the feature repository would largely accelerate this process. On the other hand, layering of detailed HistoML representations of histopathological phenotypes and digital slides provides a computational basis for quantitative analysis of histopathological phenotypes. As a result, many graph-based algorithms as well as additive models could be introduced to phenotype characterization for modelling the relationships between multi-scale histopathological features, providing more tools to systematically define histopathological phenotypes.

In addition to phenotype characterization, HistoML could contribute to computational analysis of WSIs. Used as data annotation format, HistoML could improve performance and generality of deep learning models for WSI analysis. Compared with the current annotation formats for histopathology (e.g. list or taxonomy of labels, narrative descriptions), HistoML could annotate histopathological features within histopathology data more precisely and comprehensively. Therefore, it could construct datasets covering more variations that are encountered in real-world practice, thereby developing more powerful image-processing algorithms to meet the evolving needs of pathologists and oncologists. On the other hand, HistoML could be used as an information standard to harmonize diverse image-processing algorithms and data types of WSI across research groups and programming languages, for constructing a computational pipeline of WSI analysis in a FAIR way, as Fig. 5a shows. The current pipelines^33–35^ involve mainly single-cell analysis methods; they could further incorporate the analysis methods of histopathological phenotypes by using HistoML.

There are several limitations of HistoML and Histopathology Ontology remaining to be overcome. Firstly, it is understood that there exist more types of histopathological phenotypes than HistoML Level 1 could represent explicitly. Therefore, we plan to improve HistoML by representing histopathological phenotypes of more neoplastic diseases such as cervical adenocarcinoma and insulinoma, making it more generalizable to different histopathological phenotypes while remain compatible. Secondly, morphological heterogeneity is only one aspect of intra-tumoral heterogeneity; therefore, to study the dynamic heterogeneity of cancer cells as well as the driving factors, it’s necessary to incorporate more biologically and medically meaningful features into HistoML representations. Some of the features under discussion for HistoML Level 2 are the introduction of histopathological diagnoses, which play a key role in understanding the prognostic value of histopathological phenotypes. Thirdly, due to the long time required for representing semantics of a WSI and as a result the shortage of HistoML representations, the use case presented in this paper are limited in scope. In the future, we plan to validate HistoML-powered phenotype characterization on a larger WSI dataset. As for Histopathology Ontology, despite the abundance of ontology resources that are available for reuse, some necessary histopathological features are not represented or sufficiently represented by the reference ontologies. For instance, the reference ontologies lack the detailed catalogue of descriptive cell types and histopathological phenotype terms. Therefore, more works should be conducted to add terms and metadata to Histopathology Ontology.

HistoML Level 1 is a starting point toward standardizing the representation of all histopathological features. The evolution of HistoML would be largely driven by the needs of the community. It is our hope that members of the community will support and use HistoML in the cancer research and medicine.

## Materials and Methods

### HistoML Design and Implementation

HistoML is developed through a community-based approach. We formed a group of biomedical researchers, pathologists, ontologists, computer scientists and AI scientists. The expert team of pathologists consists of the directors of pathological department from three grade “A” hospitals including the First Affiliated Hospital of Xi’an Jiaotong University, The Second Affiliated Hospital of Xi’an Jiaotong University, and Shaanxi Provincial Tumor Hospital respectively, each director with more than 30 years’ experience as well as their teams of which pathologists have more than 10 years’ experience. From February 2019 to June 2021, this group had weekly meetings to discuss the content of HistoML based on the respective needs and goals. Our group firstly decided that the aim of HistoML Level 1 is to represent histopathological features in tumor contained in digital slides. The is because on the one hand these features are key to cancer research and medicine, and on the other hand, advances of deep learning methods for image analysis have enabled extracting large amounts of histopathological features from WSIs while other technological advances, such as QH and graph-based algorithms, have improved analysis techniques of these features. Therefore, the time is ripe to systematically collect and integrate these features in a standardized format.

Then, HistoML ontology classes, reaching the group consensus, were iteratively added in HistoML. The coverage of histopathological phenotypes as well as the minimal information required to represent them in HistoML were defined according to a set of histopathology textbooks^36,37^, official guidelines^37^, as well as the pathologists’ and researchers’ knowledge and experience.

We chose to implement HistoML using OWL considering its advantages in knowledge representation, transmission and processing. As HistoML is the first OWL-based histopathology information standard, we learned from other successful practices in related fields such as BioPAX^38^, an OWL-based representation format developed for system biology. We used Protégé^39^ to create and edit HistoML. To introduce more details and encourage broad adoption, we provide the ontology specification and documentation of HistoML, available at https://histoml.com/.

### Histopathology Ontology Development

The development of Histopathology Ontology follows the principles promoted by the Open Biological and Biomedical Ontologies (OBO) Foundry (e.g. openness and collaboration). Our group worked together to construct Histopathology Ontology by reusing existing reliable ontologies, object properties and datatype properties, with the aim of systematically classifying different cell types, tissues, histopathological phenotypes etc., to cover the information HistoML represents. Existing terms from other ontologies were imported into Histopathology Ontology using Ontofox^40^.

### Development of The Exemplar HistoML representations

In this paper, we illustrate the application of HistoML by applying it to representing several histopathological features as shown in Fig. 3, Supplementary Fig. 1 and Supplementary Fig. 2. We further provide a HistoML representation to demonstrate the use of HistoML in semantic queries as shown in Fig. 4. To construct these representations, we collected five hematoxylin and eosin-stained digital slides from the First Affiliated Hospital of Xi’an Jiaotong University, including three ccRCC, one breast cancer and one papillary thyroid carcinoma. Ethical review and approval of the study was provided by the First Affiliated Hospital of Xi’an Jiaotong University. The reference number is KYLLSL-2021-420. Informed consent had been waived before the research was carried out. The data of the patients included in the study were de-identified and do not contain any protected health information or label text.

All of the exemplar HistoML representations were constructed manually by the expert team of pathologists co-working with an informatician. The representations were constructed based on the definitions of these phenotypes obtained from the pathology textbooks^36,37^, and the WHO guideline^31,41^.

### Query of Histopathological Features Encoded in HistoML

Users could query HistoML representations using SPARQL. By further mapping HistoML representations to the annotations on the slides, the visual characteristics of the features are accessible. We used scikit-image^42^ libraries to analyze visual characteristics (e.g. calculation of area and circularity).

## Supplementary information

Supplementary Information

## Data Availability

All of the HistoML representations, as well as the ontology specification and documentation of HistoML, are freely available at GitHub (https://github.com/Peiliang/HistoML) and figshare^43^.

## Code availability

The source code of this work can be downloaded from https://github.com/Peiliang/HistoML.
