# Supplementary material for: HistoML, a markup language for representation and exchange of histopathological features in pathology images
Source: Sci Data. 2022 Jul 8;9:387. doi: 10.1038/s41597-022-01505-0 (PMC9270329; doi:10.1038/s41597-022-01505-0)
Supplement: Supplementary file 1 — Supplementary Information [file 41597_2022_1505_MOESM1_ESM.pdf]

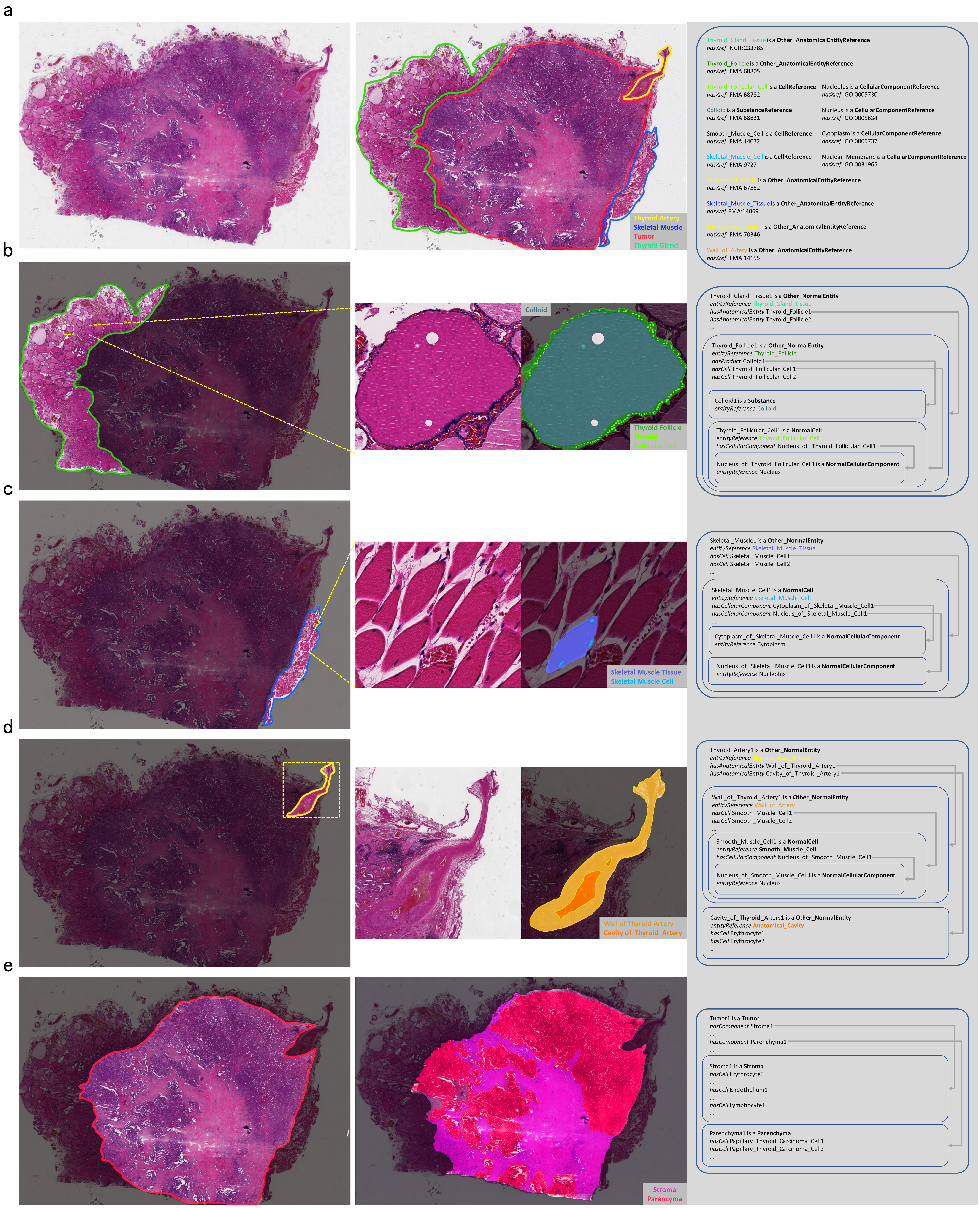

Supplementary Figure 1. Exemplar HistoML representations of different tissues as well as their parts in a whole-slide image (WSI) of papillary thyroid carcinoma. An individual of HistoML class is shown as a rounded rectangle. HistoML class names are highlighted in bold, HistoML object properties are italicized while the datatype properties are not. **(a)** An overview of this WSI in which the sample consists of four main regions including the tumor, thyroid gland tissue, skeletal muscle and thyroid artery. On the left is the original image; in the middle is the one that has been manually annotated by an expert pathologist; on the right are the individuals of **EntityReference** used to represent the histopathological entities in the WSI. **(b)** A representation of the thyroid gland tissue which consists of several thyroid follicles. In the middle are the original image of a thyroid follicle and one that has been manually annotated; on the right

is the representation. **(c)** A representation of the skeletal muscle which consists of several skeletal muscles tissues. In the middle are the original image of a skeletal muscle tissue and one that has been manually annotated; on the right is the representation. **(d)** A representation of the thyroid artery which consists of a wall and a cavity. In the middle are the original image of the thyroid artery and one that has been manually annotated and on the right is the representation. **(e)** A representation of the tumor which consists of the tumor stroma and parenchyma. In the middle is the image that has been manually annotated and on the right is the representation. The complete HistoML representations are available at https://histoml.com/ which contain descriptions of all the individual components, while space limitations permit us to show only a few in this figure.

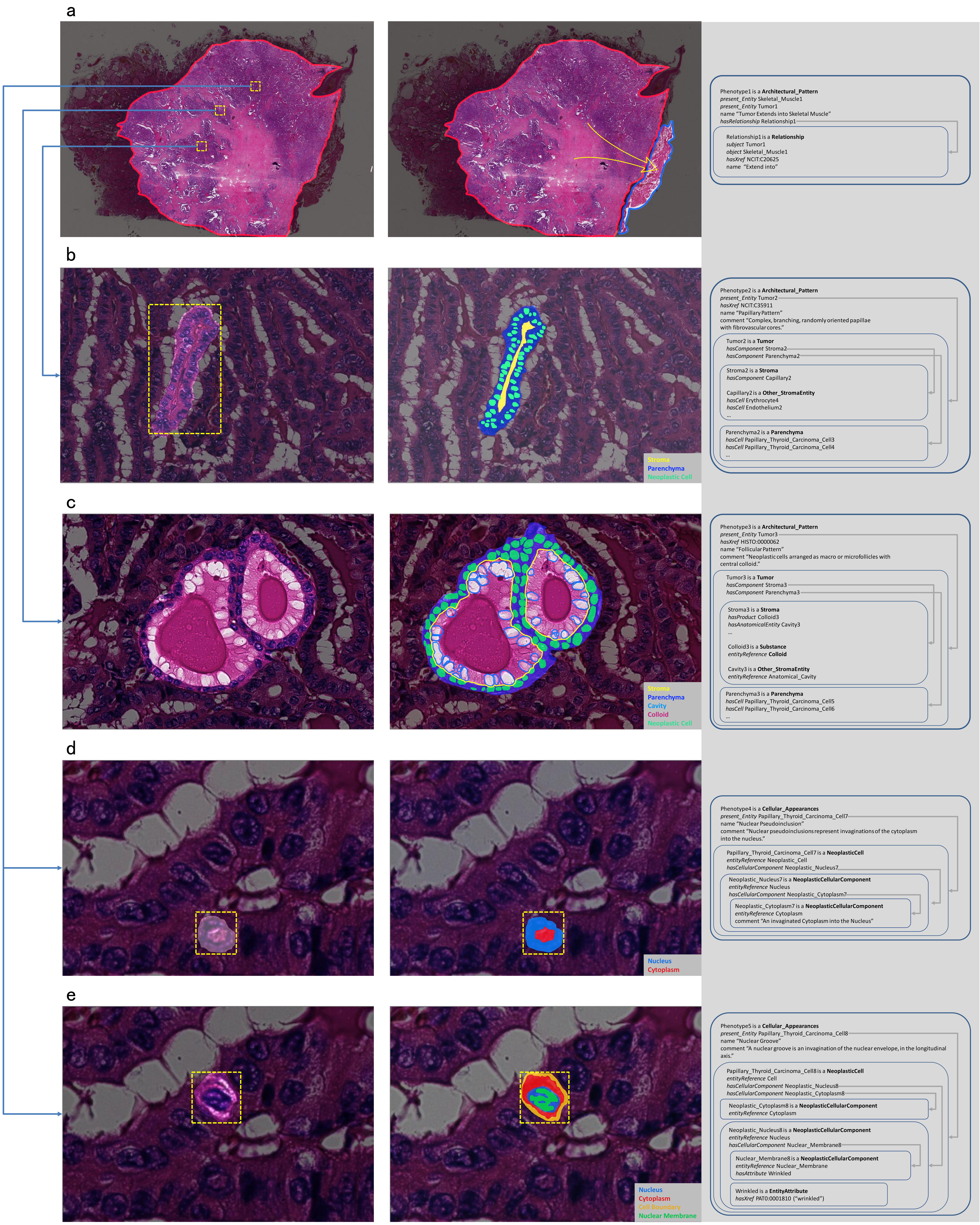

Supplementary Figure 2. Exemplar HistoML representations of histopathological phenotypes in a whole-slide image (WSI) of papillary thyroid carcinoma. An individual of HistoML class is shown as a rounded rectangle. HistoML class names are highlighted in bold, HistoML object properties are italicized while the datatype properties are not. **(a-e)** On the left are the original images; in the middle are the ones that have been manually annotated; on the right are the representations of the phenotypes as well as their individual components. The complete HistoML representations are available at https://histoml.com/ which contain descriptions of all the individual components, while space limitations permit us to show only a few in this figure.
